# Feasibility and Acceptability of the HOME Model to Promote Self-Management Among Ethnic Minority Elderly with Type 2 Diabetes Mellitus in Rural Thailand: A Pilot Study

**DOI:** 10.1089/heq.2021.0161

**Published:** 2022-08-24

**Authors:** Khanittha Pitchalard, Pawadee Wimolphan, Onnalin Singkhorn, Eva Purkey, Katemanee Moonpanane

**Affiliations:** ^1^ School of Nursing, Mae Fah Luang University, Chiang Rai, Thailand.; ^2^ School of Medicine, Queen's University, Kingston, Ontario, Canada.

**Keywords:** elderly people, ethnic minority, type 2 diabetes mellitus, rural areas, self-management

## Abstract

**Introduction:**

Ethnic minority elderly (EME) people are recognized as a vulnerable group who have higher prevalence of type 2 diabetes mellitus (T2DM) than the majority of the population. The aim of this study was to explore the feasibility, acceptability, and effect of the HOME model (Home intervention; Online monitoring; Multidisciplinary approach; and Equity and education) specifically for enhancing self-management activities, glycemic control, and satisfaction of EME with T2DM in rural areas in Thailand.

**Methods:**

In this quasi-experimental study, a single group used a pre-test and post-test, which were conducted as a pilot study to examine the effect of the HOME model.

**Results:**

Overall, 23 dyads of EME with T2DM and their family caregivers completed the 12-week intervention. They reported that the HOME model was helpful and motivating, and they reported satisfaction with the service provided. EME with T2DM showed significant reduction of blood glucose level, and significant improvement in self-management activities, happiness, and satisfaction compared with baseline. Family caregivers had also significant improvements in happiness and reported satisfaction with the HOME model.

**Conclusion:**

The primary evidence suggested that the HOME model was acceptable and feasible for EME with T2DM and their families in rural Thailand.

## Introduction

In the past three decades, the prevalence of type 2 diabetes mellitus (T2DM) has increased dramatically and has become an important global health problem.^1–4^ In 2017, ∼5 million deaths worldwide were attributable to diabetes. One of the major concerns is that more than 80% of people with T2DM live in low- and middle-income countries, accounting for 90–95% of all diagnosed cases that do not require insulin treatment.^5^ The elderly account for a significant proportion of all individuals with T2DM.^6^ According to data from the Thai Ministry of Health, about half (52.4%) of older people with T2DM fail to control their blood glucose level (HbA1c 7%)^7^ and about 200 people die each day in Thailand from T2DM.^8^ The disease burden is estimated to represent 21% per capita gross domestic product, which is about one-fifth of the average Thai economic output.^9–11^

Thailand is a country with a multiplicity of ethnicities; ethnic minorities make up 14% of the population. Ten percent of the population or ∼2.5 million live in the mountainous border areas in Northern Thailand and are commonly referred to as “hill tribe” or “chao khao” (meaning hill/mountain people or highlanders). The main six ethnic groups that are officially recognized as hill tribes are as follows: Akha, Hmong, Lahu, Lisu, Karen, and Yao. Ethnic minority people have maintained their own languages, cultures, and customs and predominantly live in rural areas, further from health care services with poor road access and public transportation. In addition to this, Apidechkul^12^ reported that over 30.0% of the ethnic minority population did not have a Thai identity card, which is used to access public services, including publicly funded health care.

In terms of health disparities, ethnic minority elderly (EME) are a particularly vulnerable group and their identities are embodied in many ways, from differences in lifestyle, as well as the experience of being stigmatized as uncivilized by the majority Thai population.^13^ With the problems of distance, language, and discrimination, their access to the Thai health care system is poor. Family caregivers are key people who support and care for EME with T2DM.^14,15^ They may also have limited understanding of health conditions related to diabetes due to low levels of education, poor health literacy, and poverty. These situations exacerbate the already vulnerable condition of EME because, in addition to T2DM, elderly people are subject to disabling complications such as cardiovascular disease, renal failure, and foot ulcers.^8,16^

Several studies have reported that family-focused and home-based interventions are associated with reduced blood glucose levels and better health outcomes for elderly people with T2DM.^14–18^ Home-based intervention involves the coordinated interaction between family caregivers and medical professionals to support patients and their caregivers.^19–21^ This can be a lifeline for the self-management that reduces anxiety, decreases transportation costs, and allows people to effectively manage diabetes at home.^22,23^ Studies have reported that home is a safe place and can be as effective as a hospital for management of elderly people with T2DM, and that people are generally satisfied with services received at home. Health education and training about the disease are important and show positive effects on both physical and psychosocial health outcomes.^8,17,24^

There is strong evidence that a home-based model is appropriate for indigenous, first nation, or tribal population patients in underserved areas to improve their clinical outcomes and quality of life.^24,25^ Thailand has successfully improved health care equity, but ethnic minority groups are mostly hidden.^12,26^ This study aims to develop a unique model based on the Innovative Care for Chronic Condition Framework [ICCCF] to improve health care system at the macro, meso, and micro levels.^27,28^ The HOME model developed for this study has the following essential elements, including Home intervention; Online monitoring; Multidisciplinary approach; and Equity and education, specifically for EME with T2DM and their family caregivers in rural areas in Thailand.

The primary purpose of this study was to explore the feasibility and the acceptability of the HOME model and the secondary purpose was to examine the effect of the HOME model specifically for enhancing self-management activities, glycemic control, and satisfaction of EME with T2DM in rural areas.

## Methods

### Study design

This is a quasi-experimental pilot study comprising a single group pre-test–post-test design to examine the effect of the HOME model on EME with T2DM in rural Thailand.

### Setting and recruitment

The setting for this pilot study was Lau Fu, a village in a remote mountain top area in Chiang Rai Province, Thailand. EME with T2DM and their family caregivers who met the inclusion criteria were recruited by a nurse at Lau Fu Subdistrict Health Promotion Hospital (SDHPH) during their monthly medical appointment. Family caregivers throughout this study were defined as adults who were identified as being a primary caregiver for the EME with T2DM regarding daily activities and diabetes management. Eligibility for participation included the following criteria: (1) EME over 60 years of age, (2) EME diagnosed with T2DM, and (3) EME living in remote areas. Participants were excluded if they had a diagnosis of dementia or evidence of significant cognitive impairment as indicated by a Mini-Mental Status Examination score of less than 24.

### Data collection and analysis

The data collection process consisted of four visits. During the first visit, the activities included screening the EME with T2DM for inclusion and exclusion criteria, consent taking procedures, and baseline assessment. Data collection was conducted by the research team and trained research assistants from baseline (week 1) to week 12 (September–November 2020). The second visit was the focus group discussion at the SDHPH meeting room. Following the self-management program, participants were divided into two groups, EME with T2DM and another group was family caregivers, to share their experiences and answer questions.

The third visit (week 4) was a telephone or LINE application, online messaging and calling application, one-on-one conversation follow-up comprising a 10–20-min seeking to identify the barriers to or opportunities for maintaining healthy behaviors and to provide further feedback. The fourth visit was a home visit by the researchers, nurse practitioners, and village health volunteers (VHVs) to teach self-management activities for controlling their blood glucose levels.

**FIG. 1.**
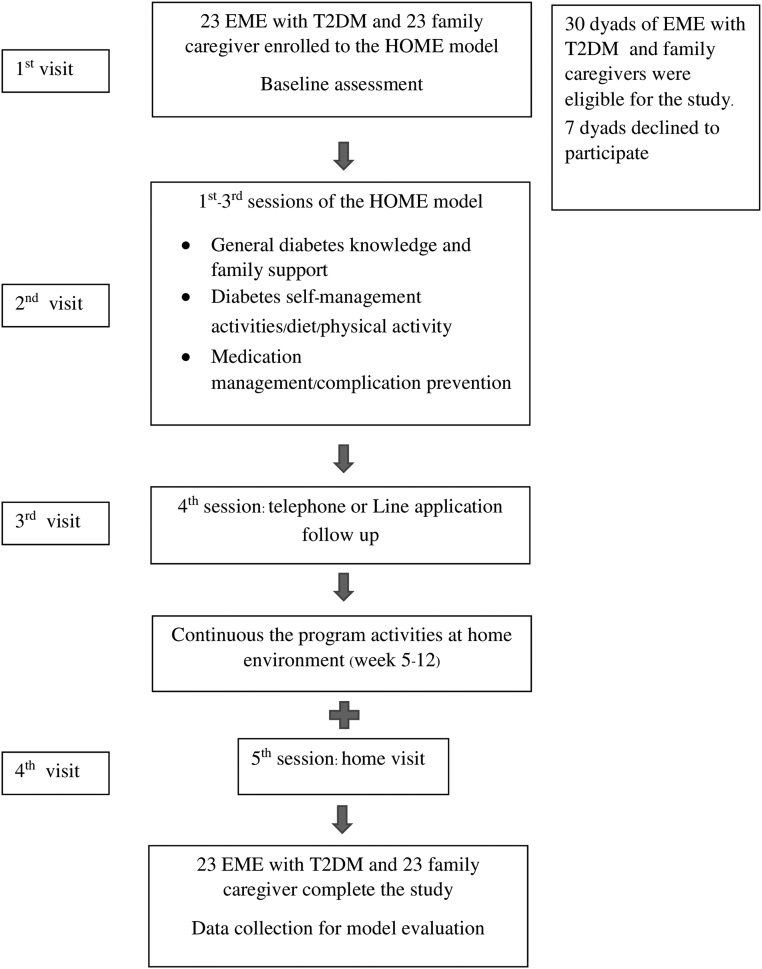
Flow chart of participant recruitment to the HOME model and activities.

At week 12, the participants were evaluated for acceptability, feasibility, outcomes, and limitations, as well as recommendation to improve the program. Continuous support and strong encouragement were given so the EME with T2DM and their family caregiver could enhance healthy behaviors and maintain behavior change (Fig. 1).

### Outcome measurements

#### Feasibility of the model

Feasibility was assessed using recruitment numbers, attendance, and completion rate of intervention sessions attended. Adherence to the intervention was facilitated by allowing EME with T2DM and their families to reschedule any session missed due to planned absences or unexpected illnesses.

#### Acceptability of the model

Acceptability was assessed at the post-intervention assessment at 12 weeks using four-questionnaire items developed for this study; responses were a three-point Likert scale (agree, neutral, and disagree). Acceptability was also assessed by semistructured interviews at the completion of the structured intervention and after the follow-up session. EME with T2DM and family caregivers were asked to describe their general experience with the HOME model.

Fasting blood sugar (FBS) level was chosen as the primary outcome because it is the main target measure when treating T2DM and can be measured when living in remote areas. FBS was collected through the nurse practitioners in SDHPH at week 1 as baseline and at week 12 as follow-up packages.

Diabetes self-management questionnaires; Thai version (DSMQ) were self-administered based on Lorig and Holman^29^ and were developed to suit the ethnic minority context. This instrument consists of 20 items encompassing knowledge around T2DM, diet, coping and stress management, self-monitoring, adherence to medication, and treatment. The T-DSMQ uses a four-point Likert scale, giving a range from 0 to 60 points. A higher score indicates greater self-management competencies. Cronbach's alpha was 0.86 of the Thai population and in this study was 0.88.

Happiness measures were developed by Fordyce,^30^ to assess a person's emotional well-being and provide an indication of a person's perceived happiness. The EME with T2DM responded to 11 questions on a 0 to 10 scale, higher scores indicating a greater level of happiness.

The client satisfaction questionnaire (CSQ) was developed by the research team to access the acceptability of the HOME model. The CSQ included five items assessing whether the intervention was perceived as appropriate, acceptable, and effective in helping to manage diabetes. The answers on a 5-point Likert scale ranged from “not at all” (0) to “extremely” (4), potential scores range from 0 to 20 points. The Cronbach's alpha coefficient for this study was 0.92.

### Data analysis

IBM^®^ SPSS statistics version 24 was used to analyze all statistical procedures for the quantitative data. The baseline demographic characteristics of age, gender, and ethnicities were described using frequency and percentage. A Wilcoxon rank-sum test was used to test the differences of study outcomes. Digital recordings were transcribed verbatim and imported into Nvivo software version 12. Qualitative content analysis using an inductive, data-driven approach was used to analyze the acceptability of data from the semistructured interviews.

### Ethical approval

Ethical approvals were obtained from the Human Ethics Research Committee, Chiang Rai Public Health Provincial Office (CRPPHO 6/2562). The purposes, risks, confidentiality, and benefits of the study were explained to the participants before they agreed to participate.

All participants had the right to refuse and withdraw at any time without penalty.

## Results

### Participant characteristics

Thirty EME with T2DM who expressed interest were screened for eligibility: twenty-three dyads of EME with T2DM and family caregivers were enrolled and completed the five sessions as well as home visit in the 12th week. The EME with T2DM had an age range between 60 and 91 years, and only three had participated in formal education. Family caregivers were predominantly female, and the majority was young adults. Selected characteristics of the EME with T2DM and family caregivers are presented in Table 1.

**Table 1.** Demographic Characteristics of the Participants

Characteristics	EME with T2DM (***n***=23)	Family caregivers (***n***=23)
Age, years		
Mean (SD)	67.87 (5.44)	47.04 (9.06)
Range, years	60–69	29–62
Sex, *n* (%)		
Female	69.57	73.91
Male	30.43	26.09
Marital status, *n* (%)		
Single	0.00	17.39
Married	82.61	73.91
Divorced/widow/separate	17.39	8.70
Education, *n* (%)		
Never attended	86.96	13.04
Primary school	13.04	43.48
Secondary school	0.00	39.13
College	0.00	4.35
Ethnicity		
Akha	34.78	34.78
Lahu	52.17	52.17
Lisu	13.04	13.04
Religion		
Buddhism	13.04	13.04
Christianity	56.52	56.52
Folk religion	30.43	30.43
Universal coverage insurance		
Yes	91.30	100.00
No	8.07	0.00
Recent fasting blood sugar, mg/dL		
<110	4.35	N/A
111–125	3043	N/A
>126	65.22	N/A

EME, ethnic minority elderly; SD, standard deviation; T2DM, type 2 diabetes mellitus.

### Development of the HOME model

The HOME model was developed through combining information and constructs drawn from the ICCCF^27^ and integrating information obtained through community consultation with 10 EME with T2DM, 10 family caregivers, 15 health care professionals, and 5 community leaders in Northern Thailand.

A 6-month planning and development period involved strategic meetings and focus group discussions around the question, “What should interventions for EME with T2DM look like if they were designed by physicians, public health officers, community leaders, VHVs, and family caregivers.” These meetings and focus groups were designed to elicit participant views on caring for EME with T2DM in rural areas. The HOME model sought to integrate cultural beliefs and values, and to be accessible to people with low literacy levels.

The goal of this intervention was to target disparities in diabetes self-management, and outcomes among ethnic communities at three levels: (1) the family and community level; (2) the district and subdistrict level; and (3) the policy level.^27^ In this model, services and activities were reviewed by five external experts in diabetes, and aimed to consider connecting links and scope of practice between regular service delivery by health care providers, local government, and VHVs (Table 2).

**Table 2.** HOME Model's Activities for Ethnic Minority Elderly with Type 2 Diabetes Mellitus and Their Family Caregivers

Session	Activities	Methods and materials	Multidisciplinary
Session 1	General diabetes knowledge and family support • Presentation of diabetes knowledge based on the National Diabetes Guidelines (2017) • Group discussion with the EME with T2DM and family caregivers about the disease and its complications, including a question-and-answer session • Provision of an education booklet to help EME with T2DM to maintain their self-management activities. This booklet was designed and tailored to the low-literacy reader, using images, pictures, symbols, and color instead of text or message	• Group discussion • Video clips • Booklet • Handout • Skill demonstration	• Physician • Nurse practitioner • Psychiatric nurse • VHVs
Session 2	Diabetes self-management activities • Review of the previous session and summary of the participant's understanding progress • Introducing the significance of self-management activities, physical activities, and stress management Diet • Introducing the categories of food and their calories • Comparing the effects of healthy and unhealthy food • Discussion on healthy eating • Use of modified ethnic food recipes Physical activity • Group discussion and answering questions about physical activities and exercise • Culturally relevant activities for exercise	• Group discussion • Video clips • Booklet • Handout • Food models • Physical activity and exercise pictures • Skill demonstrations • Problem solving	• Nurses practitioner • Physiotherapists • Dieticians • Community leader • VHVs
Session 3	Medication management • Review of the previous session and summary of the participants' self-management progress • Explanation of the significance of taking prescription medication regularly and the consequences of not doing so • Discussion of medication adherence barriers and strategies to overcome them Complication prevention • Group discussion of the main diabetic complications; prevention measures; early presentation; and consequences • Foot screening • Blood sugar interpretation	• Group discussion • Medication model and sample • Handout • Booklet • Foot model • Blood sugar testing device • Skill demonstrations • Problem solving	• Nurse Practitioner • Public health officer • VHVs • Community leader
Session 4	Telephone follow-up • Review of the previous session and connecting with the problem or concern by 10–20-min conversation on phone or LINE application • Obtaining specific complaints and analyzing the situation with the family caregiver • Providing information and guidance	• Questionnaires • Booklet • Online video clips • LINE application	• Nurse practitioner • Public health officer • VHVs
Session 5	Home visits • Review of the previous session and summary of the participant's self-management progress • Individual discussion of self-management activities and adjustment of the goal and plan • Encouraging the EME with T2DM and family caregivers with advice about how to maintain self-management activities and supportive behavior	• Questionnaires • Booklet • Online video clips • LINE application • Feedback • Supportive resources	• Nurse practitioner • Public health officer • VHVs • Community leader

VHVs, village health volunteers.

### Service feasibility

All 23 EME with T2DM and their family caregivers participated in all 5 sessions and adhered to the model and its components for 12 weeks. Suggestions included increasing the time spent for home visits, using the local to convey information about diabetes, and increasing the number of mobile eye-screening clinic sessions. Responses to the open-ended questions about the intervention were generally positive. The EME with T2DM were very pleased with the HOME model, indicating appreciation for the intervention provided, for example,

“thank you for giving information, it was good to have the intervention provided by family caregivers.” (EME 06)

“broadcasting is very helpful and thank you for reminding me to exercise.” (EME12)

“It raised my awareness and built my healthy habits, this is the time for reminding me that I am a diabetes patient and that I need to care for myself carefully.” (EME 07)

However, the negative comments were that the intervention did not cover their financial needs and participants expressed frustration when following up on an appointment, for example,

“It's hard find time to visit the SDHPH because I have to work” (EME 02)

“it would be better if some budget is provided to me while you visit.” (EME 08)

### Results of the acceptability of the model

Twenty-three EME with T2DM responded to the questionnaires and all of them found that they “agreed” the HOME model helped them to improve self-management and they were willing to participate in a similar model. If asked to participate in other HOME models, family caregiver responded “disagree” and said they were “moderately” satisfied with the HOME model because it affected their workload. This caregiver said they would not be willing to participate in another home model due to work commitments (Table 3).

**Table 3.** Acceptability to the HOME Model of the Ethnic Minority Elderly with Type 2 Diabetes Mellitus and Family Caregivers

Questionnaire item	EME with T2DM (***n***=23), ***n*** (%)			Family caregivers (***n***=23), ***n*** (%)		
	Agree	Neutral	Disagree	Agree	Neutral	Disagree
1. I have a better understanding of something new related to diabetes through the HOME model.	20 (86.96)	3 (13.04)	0	19 (82.61)	3 (13.04)	1 (4.35)
2. I found the HOME model too hard to follow.	3 (13.04)	2 (8.70)	18 (78.26)	7 (30.43)	2 (8.70)	14 (60.87)
3. I feel confident managing my diabetes.	22 (95.65)	0	1 (4.35)	20 (86.96)	0	3 (13.04)
4. I look forward to participating in a further HOME model if it is offered.	19 (82.61)	4 (17.39)	0	17 (73.91)	5 (21.74)	1 (4.35)

### Potential benefits

Table 4 shows the changes throughout the 12 weeks of the project. There was a statistically significant improvement in self-management activities, FBS level, happiness level, and satisfaction with the HOME model. The 23 EME with T2DM reported excellent support from health care providers and the community. Most of these family caregivers also reported that the HOME model improved pre-existing tension between EME with T2DM and their family caregivers. A significant change in participants was also observed in EME with T2DM satisfaction, self-management activities, and level of happiness with opportunities to consider future health care services (Table 4).

**Table 4.** Mean Self-Management Activities, Fasting Blood Sugar Level, Happiness Scores, and Satisfaction with the Model of the Ethnic Minority Elderly with Type 2 Diabetes Mellitus

	T0 (baseline), mean (SD)	T1 (12 weeks), mean (SD)	T0 to T1, ***p***-value
Fasting blood sugar, mg/dL			
EME with T2DM	130 (23.53)	113.86 (20.47)	6.673^**^
Family caregivers	N/A	N/A	—
Diabetes self-management (DSMQ) 0–60 points			
EME with T2DM	44.91 (4.64)	47.73 (3.89)	−7.230^**^
Family caregivers	N/A	N/A	N/A
Happiness (HM) (0–10 scale)			
EME with T2DM	5.00 (1.65)	6.87 (1.17)	−6.032^**^
Family caregivers	4.69 (1.29)	6.43 (1.31)	−5.509^**^
Satisfaction (CSQ) (0–20 points)			
EME with T2DM	10.08 (2.72)	15.04 (2.54)	−5.758^**^
Family caregivers	10.26 (1.62)	12.17 (2.08)	−3.447^*^

^*^ *p*<0.005, ^**^p<0.001.

CSQ, client satisfaction questionnaire; DSMQ, diabetes self-management questionnaires; HM, happiness measures.

## Discussion

The findings of this pilot study indicate that the model, derived from ICCCF^27^ and delivered by a health care professional, community key leader, and VHVs, appears to have been acceptable and feasible from the perspective of EME with T2DM.^31^

In this study, EME with T2DM are considered a vulnerable group because they experience declining physical functions and social disparities that impact their self-care abilities. Most participants also required family support. The HOME model represents partnerships between patients and their families and health care providers to raise awareness, reduce communication problems, increase responsibility for their disease management, and hopefully decrease the level of blood glucose.^17,23^

In this model, the family caregivers were highly involved in the self-management activities, helping EME with T2DM overcome barriers, maintain family function, and increase a sense of belonging, thus increasing positive emotions and well-being, which is important for EME with T2DM who lack access to care.^32^ In addition, in this pilot study, EME with T2DM stated that the HOME model led by nurses who work in this area provided a sense of security, comfort, and convenience for them and offered better options in the form of special activities that were appropriate for their culture, rituals, and social practices.^33^

A previous study found that a growing body of technology-delivered interventions was feasible and acceptable to patients with a wide range of diagnoses.^34–36^ In this study, the HOME model also provided monthly telephone follow-up or LINE application to monitor self-management activities and advise the family caregivers to maintain healthy behaviors.^37,38^ However, this study found that the technology had a better impact on the family caregivers than on EME with T2DM, given their low health literacy. If possible, diabetes technology systems should be designed to incorporate visuals, symbols, and numerals to enhance understanding.^17,39^

Community resources and local governmental organizations can also fill critical gaps in service by developing community-based supportive systems.^27^ VHVs are key components for the HOME model as they led home visits in remote areas and were available to bridge the gap between health care settings and the community. This is relevant for other developing and low-income countries as well.^20,26,40^ VHVs have the potential to reduce the burden on health facilities and VHVs are more likely to be familiar with the community and its contextual realities, which can contribute to the HOME model's feasibility and acceptability.^40,41^ Furthermore, VHVs are usually better at communicating with EME with T2DM and their family caregivers in their own language, which could help EME with T2DM overcome barriers to health care utilization.^28,31,42,43^

Collaboration with community partners also presented promising avenues for the widespread dissemination of diabetic management by local broadcasting related to diabetes knowledge, SDHPH appointments, and home visit schedules.^26,40,44^ However, one of the key challenges at the macro level is the Ministry of Health and other health care leaders must address the health care provider shortage, incentives, and other resources necessary to make improvements in the care of EME with T2DM and to create a more equitable health care system.^27,45,46^

### Limitations

This pilot study has a number of limitations that are important to consider. The small number of participants limits the generalizability of results and the statistical power. Second, we conducted a single-arm study to ascertain the feasibility and acceptability of the HOME model. Hence, this study may not be able to determine the relative benefits of the intervention. Finally, a follow-up period of 12 weeks may not be sufficient to detect improvement in glycemic levels or model sustainability.

### Recommendations

The findings from this study have multiple implications for health care professionals who deliver care for EME with T2DM. The HOME model was effective for EME with T2DM in rural areas in Thailand. An interdisciplinary home visit program should be provided regularly to improve self-management activities and glycemic outcomes, and a full-scale trial that includes a longer follow-up period is needed to determine the long-term impact of the HOME model.

## Conclusion

The pilot study evidence demonstrated that the HOME model is feasible, acceptable, and satisfactory for EME with T2DM and family caregivers. The home model also indicated significant improvements in self-management, including diabetes knowledge and treatment skills, blood sugar level, and increased satisfaction of health care delivery. Findings suggest other functional outcome measures such as HbA1c, quality of life, or diabetes knowledge could also be improved with a larger number of participants and a longer study period.

## Abbreviations Used

CSQ: client satisfaction questionnaire
DSMQ: diabetes self-management questionnaires
EME: ethnic minority elderly
FBS: fasting blood sugar
HM: happiness measures
ICCCF: Innovative Care for Chronic Condition Framework
SD: standard deviation
SDHPH: Subdistrict Health Promotion Hospital
T2DM: type 2 diabetes mellitus
VHVs: village health volunteers
